# Risk factors associated with COVID-19 infection: a retrospective cohort study based on contacts tracing

**DOI:** 10.1080/22221751.2020.1787799

**Published:** 2020-07-07

**Authors:** Tao Liu, Wenjia Liang, Haojie Zhong, Jianfeng He, Zihui Chen, Guanhao He, Tie Song, Shaowei Chen, Ping Wang, Jialing Li, Yunhua Lan, Mingji Cheng, Jinxu Huang, Jiwei Niu, Liang Xia, Jianpeng Xiao, Jianxiong Hu, Lifeng Lin, Qiong Huang, Zuhua Rong, Aiping Deng, Weilin Zeng, Jiansen Li, Xing Li, Xiaohua Tan, Min Kang, Lingchuan Guo, Zhihua Zhu, Dexin Gong, Guimin Chen, Moran Dong, Wenjun Ma

**Affiliations:** a Guangdong Provincial Institute of Public Health, Guangdong Provincial Center for Disease Control and Prevention, Guangzhou, People’s Republic of China; b Guangdong Provincial Center for Disease Control and Prevention, Guangzhou, People’s Republic of China

**Keywords:** COVID-19, attack rate, risk factors, close contact, China

## Abstract

This study aimed to estimate the attack rates, and identify the risk factors of COVID-19 infection. Based on a retrospective cohort study, we investigated 11,580 contacts of COVID-19 cases in Guangdong Province from 10 January to 15 March 2020. All contacts were tested by RT-PCR to detect their infection of SARS-COV-2. Attack rates by characteristics were calculated. Logistic regression was used to estimate the risk factors of infection for COVID-19. A total of 515 of 11,580 contacts were identified to be infected with SARS-COV-2. Compared to young adults aged 20–29 years, the infected risk was higher in children (RR: 2.59, 95%CI: 1.79–3.76), and old people aged 60–69 years (RR: 5.29, 95%CI: 3.76–7.46). Females also had higher infected risk (RR: 1.66, 95%CI: 1.39–2.00). People having close relationship with index cases encountered higher infected risk (RR for spouse: 20.68, 95%CI: 14.28–29.95; RR for non-spouse family members: 9.55, 95%CI: 6.73–13.55; RR for close relatives: 5.90, 95%CI: 4.06–8.59). Moreover, contacts exposed to index case in symptomatic period (RR: 2.15, 95%CI: 1.67–2.79), with critically severe symptoms (RR: 1.61, 95%CI: 1.00–2.57), with symptoms of dizzy (RR: 1.58, 95%CI: 1.08–2.30), myalgia (RR: 1.49, 95%CI: 1.15–1.94), and chill (RR: 1.42, 95%CI: 1.05–1.92) had higher infected risks. Children, old people, females, and family members are susceptible of COVID-19 infection, while index cases in the incubation period had lower contagiousness. Our findings will be helpful for developing targeted prevention and control strategies to combat the worldwide pandemic.

## Introduction

Since the Coronavirus Disease 2019 (COVID-19) outbreak on 31 December 2019 [1], it has hit more than 200 countries, areas or territories with 8,525,042 cases and 456,973 deaths as of 20 June 2020 [2]. World Health Organization (WHO) has declared COVID-19 as a pandemic on 11 March 2020 [3]. Owing to the effective measure taken in China, the chain of transmission has been broken and the epidemic has been under control.

Contact tracing is a major public health response to imports of rare or emerging infectious diseases. The main objectives of contact tracing are to identify potentially infected individuals before the onset of severe symptoms, and to prevent onward transmission from the secondary cases. Contact tracing has decisively contributed to the control of many infectious diseases worldwide including severe acute respiratory syndrome (SARS), Ebola virus disease, and Middle East respiratory syndrome (MERS) [4–7]. Report of the WHO-China Joint Mission on COVID-19 pointed out that China has a policy of meticulous case and contact identification for COVID-19 [8]. Previous studies using mathematical modeling also theoretically demonstrated that contact tracing and quarantine play important roles in controlling the spreading of COVID-19 [9,10]. In addition to this, contact tracing also provides a unique opportunity to investigate the epidemiological features of COVID-19.

Previous researches have analysed the data of COVID-19 patients and found some risk factors of mortality, such as older age, pre-existing cardiovascular or cerebrovascular diseases, low levels of CD3^+^CD8^+^ T-cells, high levels of cardiac troponin I, higher Sequential Organ Failure Assessment score and d-dimer [11,12]. Unfortunately, limited study has paid attention to the risk factors related to COVID-19 infection. Recent studies conducted among 1286 close contacts (98 of them were infected by SARS-CoV-2) in Shenzhen and among 2098 close contacts in Guangzhou (134 of them were infected by SARS-CoV-2) explored the risk factors for COVID-19 infection, like older age, travelling to Hubei, etc. [13,14]. Another recent study among 2761 close contact of 100 selected index cases in Taiwan identified exposure to index case with severe symptoms as a risk factor [15]. However, their limited sample size, especially the limited cases, may restrict their ability to perform detailed analysis, and reduce the power to detect significant risk factors. Additionally, findings within a single city or selected sample may restrict its ability of generalization.

In the current study, we employed a large dataset including 11,686 close contacts of COVID-19 cases (449 of them were infected) in Guangdong Province, China to estimate the attack rates, and identify risk factors for infection of COVID-19. Under the context of worldwide pandemic, understanding this issue can identify high-risk groups and provide evidence to develop targeted prevention.

## Methods

### Setting and definitions

Guangdong, a province with a large population size located in Southern China, is a place early affected by COVID-19. The first confirmed case was reported on 15 January 2020, and a total of 1361 confirmed cases were reported by 15 March 2020. Since the very early stage of COVID-19 outbreak, an intensified surveillance was implemented across Guangdong Province to detect suspected and confirmed COVID-19 cases, and their close contacts following standardized protocols released by the National Health Commission of China. Suspected and confirmed COVID-19 cases were defined based on the Diagnosis and Treatment scheme of COVID-19, and close contacts were defined by the Prevention and Control Scheme of COVID-19. These two schemes were released by the National Health Commission of China (Supplementary materials) [16,17].

### Identification and quarantine of contacts

Once a suspected or confirmed COVID-19 case was identified, the case would be reported as an index case and isolated, and the Center for Diseases Control and Prevention (CDC) will conduct a field investigation. Information of index cases was collected by clinical workers, including demographic information, exposure history, clinical symptoms, date of symptom onset, laboratory test results, and the severity. This information was directly reported to the National Internet-Based Infectious Diseases Reporting System. Information of contacts was collected by CDC using a standardized questionnaire, including general demographic characteristics, relationships with the index case, and patterns and frequency of contract. Meanwhile, their throat swabs were collected and detected by real-time reverse transcriptase polymerase chain reaction assay (RT-PCR). During the quarantine, health status of all contacts was monitored, and their throat swabs were collected every several days to test their infection status. Once they were identified with positive of severe acute respiratory syndrome coronavirus 2 (SARS-COV-2), they would be transferred to a designated hospital for diagnosis and treatment. Clinical symptoms and severity of these infected contacts were followed up and recorded by clinical workers. After 14 days’ quarantine, contacts with negative SARS-COV-2 were released.

### Statistical analysis

Categorical variables were described using percentage (%), and a Chi-square test was used to test the differences in distributions of categorical variables between index and secondary cases. If conditions for Chi-square test were not satisfied, Fisher’s exact test was used.

Attack rate was calculated as the percentage of contacts who were later confirmed to be infected with SARS-COV-2. We estimated the attack rates of contacts by gender, age, relationships to index cases (household members, relatives, social activities, etc.), transportations (flight, train, public transportation, provide car, and the Dream Cruise) where infection occurred, course of disease (incubation period, symptomatic period, and different days from symptom onset) of index cases when the contact occurred, severity of index cases (mild, moderate, severe, and critically severe), and clinical symptoms of index cases. These attack rates were calculated only using sub-datasets of the index cases and contacts with detailed information because some cases had no complete information for an estimate. Logistic regression was also conducted to estimate the risk factors of COVID-19. All data analyses were conducted by R software (version 3.5.0, R Foundation for Statistical Computing).

## Results

### General characteristics of contacts

**Figure 1.**
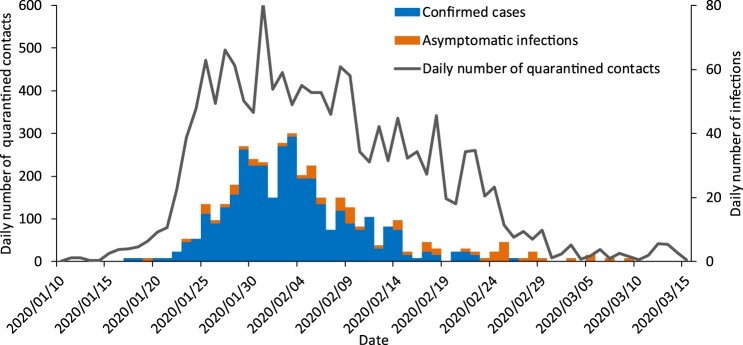
Daily numbers of quarantined contacts, and confirmed cases or asymptomatic infections identified from the quarantined contacts in Guangdong Province.

As of 15 March 2020, a total of 11,686 contacts were traced and quarantined. The first contact was identified on 10 January 2020. Contacts (*n *= 106) without key formation were excluded, and 11,580 contacts were finally included in the analysis. Figure 1 showed the daily number of quarantined contacts, which peaked (*n *= 574) on 31 January. Of total contacts, 6183 (53.4%) were males; 8419 (72.7%) were adults aged 20–59 years, and 9725 (84.0%) contacts were quarantined in centralized stations. The number of contacts occurred at home, in social activities, on transportations, and in health care settings were 4893 (40.9%), 2016 (16.8%), 3198 (26.7%) and 1348 (11.3%), respectively. Many contacts were from family members of index cases (4707, 40.7%), social activity contacts (3344, 28.9%), transportation contacts (2778, 24.0%), and health care workers (573, 4.9%) (Table 1). All contacts were linked to 1158 index cases, with a mean of 7.8 (95%CI: 7.0–8.7) close contacts per index case. The average contacts per index case varied with contact circumstances and relationships to the index cases (Table S1). The average period from exposure to quarantine was 6.4 days, and the average duration of quarantine was 9.7 days (Table S1).

**Table 1.** General characteristics of contacts to COVID-19 cases in Guangdong Province.

	*n*	%
Sex		
Male	6183	53.4
Female	5397	46.6
Age (years)		
0–9	1048	9.0
10–19	819	7.1
20–29	2420	20.9
30–39	2601	22.5
40–49	1878	16.2
50–59	1520	13.1
60–69	831	7.2
70–79	314	2.7
≥80	149	1.3
Places of quarantine		
At home	1855	16.0
Centralized stations	9725	84.0
Contact circumstances		
Family	4893	40.9
Social activities	2016	16.8
Transportation	3198	26.7
* Flight*	695	5.8
* Train*	902	7.5
Public transportation*	229	1.9
* Private car*	213	1.8
* The Dream Cruises*	64	0.5
* Unknown*	1095	9.2
Health care institutes	1348	11.3
Others	519	4.3
Relationship with index cases		
Family members	4707	40.7
*Spouse*	563	4.9
*Family members (non-spouse)*	1878	16.2
*Close relatives*	1341	11.6
*Other relatives*	925	8.0
Social activity contacts	3344	28.9
Transportation contacts	2778	24.0
Health care workers	573	4.9
Others	178	1.5
Infection spectrum of contacts		
No infection	11065	95.6
Asymptomatic infections	66	0.6
Mild confirmed cases	104	0.9
Moderate confirmed cases	300	2.6
Severe confirmed cases	31	0.2
Critically severe confirmed cases	12	0.1
Dead cases	2	<0.01

*Indicate other public transportations mainly including bus, taxi, subway, ferry, etc.

**Table 2.** Attack rates of COVID-19 in contacts with different characteristics.

Characteristics	Total contacts	Total infections	Attack Rate (%)
Age of contacts (years)			
0–9	1048	60	5.7
10–19	819	33	4.0
20–29	2420	56	2.3
30–39	2601	113	4.4
40–49	1878	56	3.0
50–59	1520	76	5.0
60–69	831	92	11.1
70–79	314	21	6.7
≥80	149	7	4.7
Sex			
Male	6183	213	3.4
Female	5397	302	5.6
Relationship to the index case			
Spouse	563	131	23.3
Family members (non-spouse)	1878	199	10.6
Close relatives	1341	94	7.0
Other relatives	925	38	4.1
Social activity contacts	3344	41	1.3
Transportation contacts	2778	10	0.3
Health care workers	573	2	0.3
Others	178	0	0.0
Contacts on different transportations			
Flight	695	6	0.8
Train	901	11	1.2
Public transportation*	229	5	2.1
Private car	213	9	4.2
The Dream Cruises	63	6	9.5
Unknown	1104	14	1.3
*Indicate other public transportations mainly including bus, taxi, subway, ferry, etc.			
Disease history of confirmed index cases#			
Incubation period	2211	72	3.3
Symptomatic period	5904	411	7.0
Contacts to the index cases at different time (days to the symptom onset)*			
≤−5	522	9	1.7
−4 to −3	283	6	2.1
−2 to −1	974	25	2.5
0	1020	61	5.6
1–2	1036	81	7.3
3–4	865	97	10.1
5–6	702	61	8.0
7–8	371	31	7.7
9–10	223	16	6.7
11–12	106	6	5.4
13–14	109	4	3.5
15–16	188	10	5.1
≥17	265	11	4.0
Clinical severity of index case			
Mild	1244	57	4.6
Moderate	5637	344	6.1
Severe	812	52	6.4
Critically severe	371	28	7.5

*Minus number indicates days before the symptom onset, plus number indicates the days after the symptom onset in confirmed cases, and zero indicates the day of symptom onset. In order to precisely estimate the contacting time, only the pairs with only one index case and one secondary case were included.

### Attack rates of COVID-19

Until 15 March 515 (4.4%) contacts were identified to be infected with SARS-COV-2. The attack rates varied by age groups with the highest for the group aged 60–69 years (11.1%), and the lowest for the group of 20–29 years (2.3%) (Table 2). The attack rate of children <10 years was 5.7%, and the attack rates were higher in children whose index cases aged 30–39 years (8.5%), and 50–59 years (7.0%) (Table S3).

We also observed a higher attack rate in females (5.6%) than in males (3.5%). In addition, contacts having a close relationship with index cases had higher attack rate (attack rate: 23.3% for spouse; 10.6% for non-spouse family members; 7.0% for close relatives; 4.1% for other relatives, 1.3% for social activity contacts, etc.). Different attack rates also occurred in various transportations where infection occurred. Attack rates were 0.8% on flight, 1.2% on train, 2.1% on public transportation, 4.2% on private car, and 9.5% on the Dream Cruise.

When considering the time contacting with the index cases, attack rates were 3.3% and 7.0% when contacts occurred in the index cases’ incubation period and symptomatic period. In detail, attack rate increased from five days prior to the symptom onset of index cases (1.7%), to a peak during 3–4 days (10.1%) after onset, and then decreased to 4.0% after 17 days of the onset. In addition, attack rates increased from 4.6% for the contacts of mild cases to 7.5% for the contacts of critically severe cases. Table S2 shows attack rates for the contacts of index cases with different clinical symptoms, and higher attack rates were observed in index cases with dyspnea (11.2%), dizzy (10.6%), muscle soreness (10.4%), and shortness of breath (10.0%).

### Risk of infection for COVID-19

**Figure 2.**
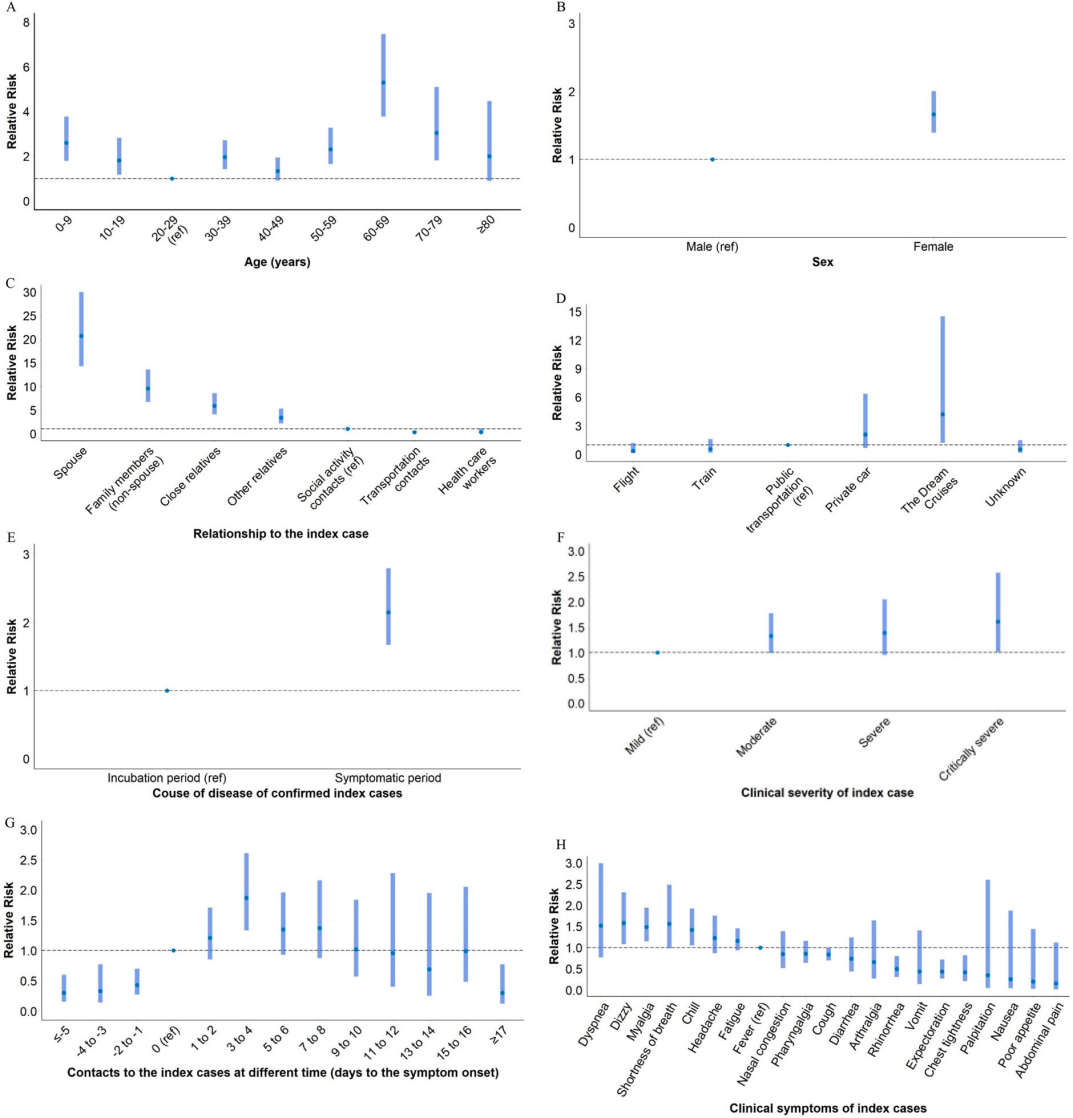
Infected risks of COVID-19 in contacts with different characteristics. (A) In contacts with different ages; (B) In males and females; (C) In contacts who had different relationships to the index case; (D) In contacts exposed to the index cases on different transportations; (E) In contacts exposed to the index cases at different time; (F) In contacts exposed to the index cases in different course of disease; (G) In contacts exposed to the index cases with different clinical severity; (H) In contacts exposed to the index cases with different clinical symptoms. Adjusted for age and/or sex.

Compared with people aged 20–29 years, children <10 years (RR: 2.59, 95%CI: 1.79–3.76) and children aged 10–19 (RR: 1.81, 95%CI: 1.17–2.81) had higher risk to be infected with COVID-19 (Figure 2A). The risks were also higher in people aged 30–39 years (RR: 1.96, 95%CI: 1.41–2.71), 50–59 years (RR: 2.30, 95%CI: 1.65–3.27), 60–69 years (RR: 5.29, 95%CI: 3.76–7.46) and 70–79 years (RR: 3.03, 95%CI: 1.81–5.08). Moreover, young adults (aged 30–39 years), whose index cases aged <20 years, 30–39 years, and 50–69 years, had higher infected risk (Table S4). We also observed a higher risk in females than in males (RR: 1.66, 95%CI: 1.39–2.00) (Figure 2B). In addition, people having close relationship with index cases encountered higher risk to be infected (RR and 95%CI: 20.68 [14.28–29.95] for spouse; 9.55 [6.73–13.55] for non-spouse family members; 5.90 [4.06–8.59] for close relatives; 3.37 [2.15–5.28] for other relatives) (Figure 2C). In terms of the infected risk in transportations, we did not observe significant difference across various transportations except in the Dream Cruises (RR: 4.19, 95%CI: 1.21–14.50) (Figure 2D).

When considering time contacting with index cases, the risk of exposure to index cases in the symptomatic period was higher than in the incubation period (RR: 2.15, 95%CI: 1.67–2.79) (Figure 2E). More specifically, the infected risk increased from five-plus days prior to the symptom onset of index cases (RR: 0.30, 95%CI: 0.15–0.60), to a peak during 3–4 days (RR: 1.87, 95%CI: 1.33–2.61) after onset, and then decreased to 0.30 (95%CI: 0.12–0.77) after 17 days of the onset (Figure 2F). Moreover, contact with index cases with critically severe symptoms was associated with a higher infected risk (RR: 1.61, 95%CI: 1.00–2.57) (Figure 2G). Figure 2H shows the infected risk for the contacts of index cases with different clinical symptoms compared to fever, and there were higher risks in index cases with dizzy (RR: 1.58, 95%CI: 1.08–2.30), myalgia (RR: 1.49, 95%CI: 1.15–1.94), and chill (RR: 1.42, 95%CI: 1.05–1.92).

## Discussion

After reporting the first case on 15 January 2020, Guangdong Provincial government mobilized enormous resources to respond to the COVID-19 epidemic. More than 11,000 close contacts of COVID-19 were traced and quarantined. One-third of the total cases reported in Guangdong Province were identified from these contacts, which indicate that contact tracing strategy has played an important role in containing the spreading of COVID-19. The analysis of index cases and their close contacts provides insight into the attack rates and risk factors of infection for COVID-19.

We found that attack rates were higher in the elderly with the highest in the group aged 60–69 years, and logistic regression demonstrated the statistical significance. These findings are consistent with the results for SARS in Beijing [7]. Recent studies also reported that elderly contacts were more likely to encounter COVID-19 infection [13,14]. However, another recent article in Taiwan did not observe significant higher infected risk of elderly contacts, which may ascribe its insufficient sample size [15]. Our findings thus confirmed the greater vulnerability of the elderly. Those contacts aged 60–69 years could have more physical activities than older people, which may cause closer contact with index case for a longer period [7]. Meanwhile, the immunity of the age may be weaker than younger adults, making them more susceptible to infection. Therefore, more efforts are needed to protect the elderly from the infection of COVID-19.

The susceptibility of children to COVID-19 is controversial [8,18]. Clinical data of COVID-19 showed much lower percentage of children aged <10 years [19,20]. A recent systematic review considering literatures of COVID-19 in children pointed out that children cases are usually less severe than adult cases, and more children cases are asymptomatic infection, which makes them less opportunity to be tested and identified [21]. However, we found the higher infected risk of COVID-19 in children <10 years that their RR were larger than contacts aged 10–59 years, which indicates that children were also susceptible to COVID-19. Furthermore, we observed a higher attack rate in children whose index cases aged 30–39 and 50–59 years. Although limited sample size may cause insignificant RR, our results still implicated that the children may be mainly infected by their parents and grandparents. Two recent studies reported consistent results with our study [13,22]. For instance, Dong et al. analysed 2143 pediatric COVID-19 patients across China, and found that children were susceptible to COVID-19 [22]. Additionally, young adults (30–39 years) were more likely to be infected by children aged < 20 years, their peers aged 30–39 years, as well as people aged 50–69 years. These findings may be attributed to the status that young adults are the primary caregivers once their children and parents got sick, and they are also the individuals who have many social activities with their peers. These findings suggested that people should performed strict personal protection both at home and in public places. Compared with previous studies, our study prospectively collected data based on contacts tracing, which had explicit temporality for causal inference and reduced recall bias, and therefore provide more reliable evidence. Our finding is helpful for preventing people from being infected with COVID-19.

We observed that female contacts were more likely to be infected by SARS-CoV-2 than male contacts, which is consistent with previous studies [13,14]. For example, a recent study conducted in Guangzhou also found higher attack rates in females than in males [14]. This difference in attack rate between sex may be due to several reasons: (1) females play predominant roles as caregivers within the family and may have closer contact and longer contact period with the index cases [23]; (2) females comprise a large proportion of health care workers [24]. Therefore, our findings suggest more prevention measures specifically implemented to protect females from infection during the epidemic of COVID-19.

We observed that the relationships between contacts and index cases significantly affected the infected risks. Compared with the social activity contacts, the risk of being infected was more than 20 times higher among the spouse and more than nine times higher among other family members, which was consistent with previous studies on SARS and H1N1 [7,25]. A newly published research also found that more infections were acquired in household [15]. Family members are more likely to have closer contact with index case for a longer contact period with the shorter distance. Another possible reason is that family members may have some certain linkage with index cases in living habits which may cause higher predisposition in infection than other close contacts. Unfortunately, individuals commonly take protective measures in public place like washing hands and wearing mask, but neglect personal protection at home. This indicates the necessity for public to pay attention to personal protective at home especially when family members develop symptom or have travel history of epidemic areas.

We also compared attack rates occurred on different transportations, and found lower attack rates occurred on trains or flights. This result indicates that the possibility of transmission of SARS-COV-2 on flight and train was low, which may be related to the advanced air purification system and sanitation in these transportations. However, after controlling for age and sex, the results of logistic regression did not find significant difference across various transportations except in the Dream Cruises. The insignificance may be attributed to the limited sample size and the risk difference may actually exist. Future studies with a larger sample should be conducted to explore this issue and provide evidence to guide the development of prevention in transportations.

Although previous studies reported that both asymptomatic and symptomatic cases could infect other persons [26–28], the differences in contagiousness at different phases of COVID-19 remain unclear. Our study shows the contagiousness peaked during 3–4 days after symptom onset, which is consistent with previous studies, which showed higher virus shedding during several days after the onset of symptoms [29–31]. For example, To et al. found that salivary viral load in COVID-19 cases was highest during the first week after symptom onset, and the viral RNA was detected 25 days after symptom onset [31]. In addition, we found contacts before the symptom onset could also lead to infection, which indicates the transmission of COVID-19 in incubation period. Although viral shedding before symptom onset is still limited, Zou et al. reported an asymptomatic patient who had a similar amount of virus to those symptomatic cases [30]. Another study conducted in children also detected positive virus before the onset of symptoms in several children cases [20]. These findings suggested COVID-19 could be transmitted before the onset of symptoms.

The present study found that severe index cases could cause higher attack rates than mild cases. In addition, compared with cases with fever, dizzy, myalgia, and chill caused higher infected risks to their contacts, while cases with rhinorrhea, expectoration, and chest tightness caused lower infected risks. To et al.’s study showed higher virus load in specimens of severe patients than mild patients [31], which verified our findings. However, studies are needed to detect the virus load in cases with different clinical symptoms for assessing their contagiousness.

This study has several strengths. First, our study includes the largest number of close contacts of COVID-19 to date. Second, our study is a retrospective cohort study, which provides information with explicit temporality for causal inference, and the recall bias was reduced. Third, we estimated the attack rates and infected risks for different contacts, which is helpful for identifying susceptible groups to develop specific protection. Fourth, we estimated the contagiousness across the course of COVID-19.

Some limitations also need to be noted. First, although we used a large dataset with more than 10,000 of contacts, the sample size of cases was limited in some subgroups, which may lead to insufficient power to identify the statistical significance. Second, a number of asymptomatic infections may be missed and their close contacts cannot be identified. Third, since the imperfect sensitivity of the RT-PCR test, some potential infections among close contacts may be missed. Fourth, the data were collected by a variety of epidemiological investigation groups across Guangdong Province. Despite using the same protocol, the implementation may have inconsistence and some noise may be introduced.

## Conclusions

Children, old people, females, and family members are susceptible to be infected with COVID-19, while index cases in the incubation period had lower contagiousness. Our findings will be helpful for developing targeted prevention and control strategies to combat the worldwide pandemic.

## Supplementary Material

Supplementary_materials20200518.docx
