# Supplementary material for: Risk factors associated with COVID-19 infection: a retrospective cohort study based on contacts tracing
Source: Emerg Microbes Infect. 2020 Jul 7;9(1):1546–53. doi: 10.1080/22221751.2020.1787799 (PMC7473290; doi:10.1080/22221751.2020.1787799)
Supplement: Supplementary_materials20200518.docx [file TEMI_A_1787799_SM0329.docx]

**Supplementary materials**

**Risk factors associated with COVID-19 infection: a retrospective cohort study based on contacts tracing**

Tao Liu^1^*, Wenjia Liang^2^*, Haojie Zhong^2^*, Jianfeng He^2^*, Zihui Chen^1^*, Guanhao He^1^*, Tie Song^2^, Shaowei Chen^1^, Ping Wang^1^, Jialing Li^2^, Yunhua Lan^2^, Mingji Cheng^2^, Jinxu Huang^2^, Jiwei Niu^2^, Liang Xia^2^, Jianpeng Xiao^1^, Jianxiong Hu^1^, Lifeng Lin^2^, Qiong Huang^2^, Zuhua Rong^1^, Aiping Deng^2^, Weilin Zeng^1^, Jiansen Li^2^, Xing Li^1^, Xiaohua Tan^2^, Min Kang^2^, Lingchuan Guo^1^, Zhihua Zhu^1^, Dexin Gong^1^, Guimin Chen^1^, Moran Dong^1^, Wenjun Ma^1†^

1. Guangdong Provincial Institute of Public Health, Guangdong Provincial Center for Disease Control and Prevention, Guangzhou, China.

2. Guangdong Provincial Center for Disease Control and Prevention, Guangzhou, China.

*These authors contributed equally to this work

†The author was corresponding author.

Correspondence to:

Dr.Wenjun Ma, Guangdong Provincial Institute of Public Health, Guangdong Provincial Center for Disease Control and Prevention, Guangzhou 511430, China

mawj@gdiph.org.cn

**1.1 Definitions of suspect and confirmed cases**

Considering both the following epidemiological history and clinical manifestations:

Epidemiological history

(1) History of travel to or residence in Wuhan and its surrounding areas, or in other communities where cases have been reported within 14 days prior to the onset of the disease;

(2) In contact with novel coronavirus infected people (with positive results for the nucleic acid test) within 14 days prior to the onset of the disease;

(3) In contact with patients who have fever or respiratory symptoms from Wuhan and its surrounding area, or from communities where confirmed cases have been reported within 14 days before the onset of the disease; or

(4) Clustered cases (2 or more cases with fever and/or respiratory symptoms in a small area such families, offices, schools etc. within 2 weeks).

Clinical manifestations

(1) Fever and/or respiratory symptoms;

(2) The aforementioned imaging characteristics of novel coronavirus pneumonia (SARS-COV-2);

(3) Normal or decreased WBC count, normal or decreased lymphocyte count in the early stage of onset.

A suspect case has any of the epidemiological history plus any two clinical manifestations or all three clinical manifestations if there is no clear epidemiological history.

Confirmed cases

Suspect cases with one of the following etiological or serological evidences:

(1) Real-time fluorescent RT-PCR indicates positive for new coronavirus nucleic acid;

(2) Viral gene sequence is highly homologous to known new coronaviruses.

(3) SARS-COV-2 virus specific IgM and IgG are detectable in serum; SARS-COV-2 virus specific IgG is detectable or reaches a titration of at least 4-fold increase during convalescence compared with the acute phase.

**1.2 Definition of close contacts to the index case**

A contact is a person who experienced any one of the following exposures during the 2 days before and the 14 days after the onset of symptoms of a probable or confirmed case:

(1) Face-to-face contact with a probable or confirmed case within 1 meter and for more than 15 minutes.

(2) Direct physical contact with a probable or confirmed case.

(3) Direct care for a patient with probable or confirmed COVID-19 disease without using proper personal protective equipment. or

(4) Other situations as indicated by local risk assessments.

*Note: for confirmed asymptomatic cases, the period of contact is measured as the 2 days before through the 14 days after the date on which the sample was taken which led to confirmation.*

**1.3 Definition of asymptomatic infection**

Asymptomatic infection was defined as those whose specimens are detected with positive of SARS-COV-2 virus, but have no clinical symptoms including fever, cough, etc.

Table S1. Characteristics of contact tracing and quarantine of COVID-19 in Guangdong Province

|  | Parameter (95%CI) |
| --- | --- |
| Average number of contacts linked to one index case* | 7.8 (7.0, 8.7) |
| **Contacts divided by contact circumstances** |  |
| Family | 5.3 (4.7, 6.0) |
| Social activity | 12.5 (10.2, 14.7) |
| Transportation | 10.8 (9.3, 12.2) |
| Health care institute | 22.8 (17.4, 28.2) |
| Others | 8.0 (1.9, 14.1) |
| **Contacts divided by relationship to index cases** |  |
| Household member | 5.1 (4.2, 5.9) |
| Social activity contact | 14.6 (12.3, 16.8) |
| Transportation contact | 12.7 (10.9, 14.5) |
| Health care worker | 15.9 (11.9, 19.9) |
| Others | 11.2 (-2.0, 24.4) |
| Average days from exposure to quarantine | 6.4 (6.2, 6.6) |
| Average days of quarantine | 9.7 (9.6, 9.8) |

*: The parameter was estimated in the total contacts.

Table S2. Attack rates of COVID-19 in contacts of index cases with different clinical symptoms

| Clinical symptoms of index cases | Number of contacts | Total infections | Attack Rate (%) |
| --- | --- | --- | --- |
| Total | 8115 | 483 | 6.0 |
| Dyspnea | 89 | 10 | 11.2 |
| Dizzy | 322 | 34 | 10.6 |
| Myalgia | 760 | 79 | 10.4 |
| Shortness of breath | 219 | 22 | 10.0 |
| Chill | 598 | 54 | 9.0 |
| Headache | 469 | 39 | 8.3 |
| Fatigue | 1552 | 122 | 7.9 |
| Fever | 4989 | 335 | 6.7 |
| Nasal congestion | 305 | 18 | 5.9 |
| Pharyngalgia | 945 | 55 | 5.8 |
| Cough | 3996 | 225 | 5.6 |
| Diarrhea | 320 | 16 | 5.0 |
| Arthralgia | 117 | 5 | 4.3 |
| Rhinorrhea | 525 | 19 | 3.6 |
| Vomit | 85 | 3 | 3.5 |
| Expectoration | 595 | 18 | 3.0 |
| Chest tightness | 315 | 9 | 2.9 |
| Palpitation | 38 | 1 | 2.6 |
| Nausea | 65 | 1 | 1.5 |
| Poor appetite | 79 | 1 | 1.3 |
| Abdominal pain | 121 | 1 | 0.8 |
| Chest pain | 7 | 0 | 0.0 |

Adjusted for age and sex.

Table S3. Attack rates (%) of COVID-19 in contacts who contacted to the index cases with different ages

|  | Age (years) of the index case (number of infections/number of contacts, %) | | | | | | | | |
| --- | --- | --- | --- | --- | --- | --- | --- | --- | --- |
| Age of contact (years) | 0-9 | 10–19 | 20–29 | 30–39 | 40–49 | 50–59 | 60–69 | 70–79 | ≥80 |
| 0-9 | 0 (0.0) | 0 (0.0) | 1/88 (1.1) | 17/201 (8.5) | 3/94 (3.2) | 8/114 (7.0) | 3/106 (2.8) | 0 (0.0) | 0 (0.0) |
| 10–19 | 0 (0.0) | 1/24 (4.2) | 0 (0.0) | 5/101 (5.0) | 9/113 (8.0) | 2/53 (3.8) | 1/69 (1.4) | 2/29 (6.9) | 0 (0.0) |
| 20–29 | 0 (0.0) | 0 (0.0) | 6/357 (1.7) | 4/249 (1.6) | 9/266 (3.4) | 13/259 (5.0) | 1/149 (0.7) | 1/71 (1.4) | 2/26 (7.7) |
| 30–39 | 4/39 (10.3) | 2/27 (7.4) | 4/254 (1.6) | 25/361 (6.9) | 2/259 (0.8) | 21/284 (7.4) | 19/273 (7.0) | 2/54 (3.7) | 0 (0.0) |
| 40–49 | 1/11 (9.1) | 2/42 (4.8) | 1/185 (0.5) | 2/197 (1.0) | 12/260 (4.6) | 3/199 (1.5) | 5/167 (3.0) | 11/61 (18.0) | 1/27 (3.7) |
| 50–59 | 0 (0.0) | 0 (0.0) | 4/197 (2.0) | 9/144 (6.3) | 4/114 (3.5) | 24/271 (8.9) | 7/152 (4.6) | 4/45 (8.9) | 1/27 (3.7) |
| 60–69 | 0 (0.0) | 2/12 (16.7) | 1/47 (2.1) | 13/103 (12.6) | 5/59 (8.5) | 12/86 (14.0) | 33/168 (19.6) | 7/40 (17.5) | 0 (0.0) |
| 70– 79 | 0 (0.0) | 0 (0.0) | 0 (0.0) | 2/19 (10.5) | 4/38 (10.5) | 1/41 (2.4) | 3/47 (6.4) | 5/39 (12.8) | 2/6 (33.3) |
| ≥80 | 0 (0.0) | 0 (0.0) | 0 (0.0) | 0 (0.0) | 1/25 (4.2) | 3/25 (12.0) | 3/13 (23.1) | 0 (0.0) | 0 (0.0) |

Table S4. Relative risk (RR) of COVID-19 in contacts who contacted to the index cases with different ages

|  | Age of the index case (years) | | | | | | | | |
| --- | --- | --- | --- | --- | --- | --- | --- | --- | --- |
| Age of contact (years) | 0-9 | 10–19 | 20–29 | 30–39 | 40–49 | 50–59 | 60–69 | 70–79 | ≥80 |
| 0-9 | - | - | 0.36 (0.04, 3.52) | 2.81 (0.80, 9.84) | Reference | 2.31 (0.59, 8.97) | 0.89 (0.17, 4.52) | - | - |
| 10–19 | - | 0.51 (0.06, 4.23) | - | 0.60 (0.19, 1.86) | Reference | 0.45 (0.09, 2.17) | 0.17 (0.02, 1.37) | 0.86 (0.17, 4.21) | - |
| 20–29 | - | - | 0.51 (0.18, 1.45) | 0.49 (0.15, 1.62) | Reference | 1.61 (0.67, 3.88) | 0.21 (0.03,1.67) | 0.44 (0.05, 3.55) | 2.52 (0.51, 12.42) |
| 30–39 | 12.77 (2.23, 72.87) | 10.25 (1.37, 76.71) | 2.11 (0.38. 11.66) | 8.77 (2.05, 37.45) | Reference | 10.28 (2.38, 44.39) | 8.39 (1.93, 36.50) | 3.97 (0.54, 28.96) | - |
| 40–49 | 2.05 (0.24. 17.41) | 1.07 (0.23, 4.97) | 0.11 (0.01, 0.85) | 0.21 (0.05, 0.97) | Reference | 0.31 (0.09, 1.11) | 0.63 (0.22, 1.83) | 4.37 (1.82, 10.51) | 0.75 (0.09, 6.05) |
| 50–59 | - | - | 0.51 (0.12, 2.08) | 1.65 (0.49, 5.55) | Reference | 2.47 (0.83, 7.31) | 1.17 (0.33, 4.11) | 2.44 (0.58, 10.31) | 0.90 (0.10, 8.47) |
| 60–69 | - | 2.26 (0.38, 13.48) | 0.25 (0.03, 2.27) | 1.53 (0.51, 4.54) | Reference | 1.85 (0.61, 5.61) | 2.56 (0.94, 6.93) | 2.24 (0.65, 7.68) | - |
| 70– 79 | - | - | - | 1.05 (0.17, 6.39) | Reference | 0.21 (0.02, 2.00) | 0.57 (0.12, 2.71) | 1.19 (0.29, 4.85) | 3.95 (0.53, 29.25) |
| ≥80 | - | - | - | - | Reference | 2.78 (0.26, 30.21) | 5.71 (0.48, 68.55) | - | - |

Adjusted for sex.
